# Factors associated with spinal fixation mechanical failure after tumor resection: a systematic review and meta-analysis

**DOI:** 10.1186/s13018-022-03007-6

**Published:** 2022-02-20

**Authors:** Zhenyu Cai, Yongzhao Zhao, Xiaodong Tang, Rongli Yang, Taiqiang Yan, Wei Guo

**Affiliations:** Musculoskeletal Tumor Center, Peking University People’s Hospital, No. 11 Xizhimen South Street, Xicheng District, Beijing, 100044 China

**Keywords:** Spinal tumor resection, Spinal fixation mechanical failure, Risk factors, Tumor level, Cage subsidence, Radiotherapy, Meta-analysis

## Abstract

**Background:**

No available meta-analysis has been published that systematically assessed spinal fixation mechanical failure after tumor resection based on largely pooled data. This systematic review and meta-analysis aimed to investigate the spinal fixation failure rate and potential risk factors for hardware failure.

**Methods:**

Electronic articles published between January 1, 1979, and January 30, 2021, were searched and critically evaluated. The authors independently reviewed the abstracts and extracted data on the spinal fixation failure rate and potential risk factors.

**Results:**

Thirty-eight studies were finally included in the meta-analysis. The pooled spinal fixation mechanical failure rate was 10%. The significant risk factors for hardware failure included tumor level and cage subsidence. Radiotherapy was a potential risk factor.

**Conclusion:**

The spinal fixation mechanical failure rate was 10%. Spinal fixation failure is mainly associated with tumor level, cage subsidence and radiotherapy. Durable reconstruction is needed for patients with these risk factors.

## Introduction

The spine is a common site of musculoskeletal tumors, and spinal tumor patients must undergo spinal surgery to relieve neural compression, control local tumors and prolong survival [1, 2]. After resecting the tumor, internal fixation is used to attain spinal stability [3, 4]. Given the increased survival of patients, there is a growth trend of fixations experiencing failure. Spinal hardware failure could cause spinal instability and decrease the quality of life of patients [5–10]. To avoid the mechanical failure of spinal fixation, it is important to study factors related to the current situation.

Although some studies [7, 11–16] on spinal fixation mechanical failure after tumor resection have been published, some questions remain unanswered. First, most current studies describe only the rate of spinal hardware failure and the potential risk factors based on clinical experience, and these studies lack statistical risk factor analyses [13, 16, 17]. Second, statistical analysis was only performed in a few studies, and the population of included patients was small, which may affect the results [3, 4, 18]. In addition, not all studies included vertebral location [3–5, 11] as a risk factor. Therefore, to better guide clinical therapy, a meta-analysis is urgently needed to investigate the factors associated with spinal fixation mechanical failure.

## Materials and methods

### Search strategy

A comprehensive literature search was performed using the PubMed, EMBASE, Web of Science, and Cochrane Library databases for studies published between January 1, 1979, and January 30, 2021. The following *MeSH* terms and their combinations were searched: ((Spine[*MeSH* Terms]) AND (((Neoplasms[*MeSH* Terms]) OR (Sarcoma[*MeSH* Terms])) OR (Carcinoma[*MeSH* Terms]))) AND ((((instrumentation failure) OR (fixation failure)) OR (hardware failure)) OR (Rod fracture)). Two authors independently reviewed the titles and abstracts to screen and extract relevant articles.

### Selection criteria

The PICOS criteria for inclusion and exclusion were as follows:

- P (participants): Studies of spinal tumor surgery were included.
- I and C (intervention and control): Studies in which spinal tumor patients received tumor resection and spinal fixation were included. If some studies included partially duplicated patients, only the studies that used large and advanced data were included.
- O (outcome): Studies that included patients with spinal fixation mechanical failure with or without the following clinicopathologic factors were included: sex, age, chemotherapy, radiotherapy, tumor histology, location, surgical approach, number of vertebrae resected, rod diameter, constructed length and cage subsidence. For risk factor analysis, only the studies reporting fixation failure rates stratified by each risk factor were included. When a study reported the results on different subpopulations, we regarded data from the subpopulations as separate studies in the meta-analysis.
- S (study type): Research articles published between January 1, 1979, and January 30, 2021, were included. All review papers, meta-analyses, and case reports were excluded.

### Quality assessments

The quality of each eligible study was rated independently by two reviewers using the modified Newcastle–Ottawa scale 27. A score of 0–9 was assigned to each study.

### Data extraction

A data collection sheet was developed to record the level of evidence, study quality, available outcomes, and risk factors. Two investigators independently extracted data from these studies. If the variable was divided into dichotomous subgroups, data from the two subgroups were included regardless of the cutoff value. If the variable was divided into polytomous rather than dichotomous subgroups, only the data of subgroups in both ends were included.

### Statistical analysis

The analyses were performed using Stata 14.0 (StataCorp, College Station, TX, USA). We used a random-effects model to produce a pooled overall estimate for the spinal fixation failure rate with Stata 14.0. The OR was used to compare dichotomous variables. All results were reported with 95% CI. Statistical heterogeneity between studies was assessed using the Chi-square test and quantified using the *I*^2^ statistic. If *p* < 0.1 and *I*^2^ ≥ 50%, the random-effects model was used to merge the ORs. If *p* > 0.1 and *I*^2^ < 50%, the fixed-effect model was used to merge the OR values. When OR > 1, the factors were accepted as risk factors resulting in fixation failure. When OR < 1, the factors were accepted as protective factors avoiding fixation failure. If significant heterogeneity was noted, an increased quantity of included studies was necessary.

### Sensitivity analysis and publication bias

Sensitivity analysis was performed to evaluate whether the results of the meta-analysis changed after the removal of any one study. To assess the presence of publication bias, we used funnel plots and Egger’s test. A value of *p* < 0.05 indicated statistically significant publication bias.

## Results

### Study characteristics

**Fig. 1:**
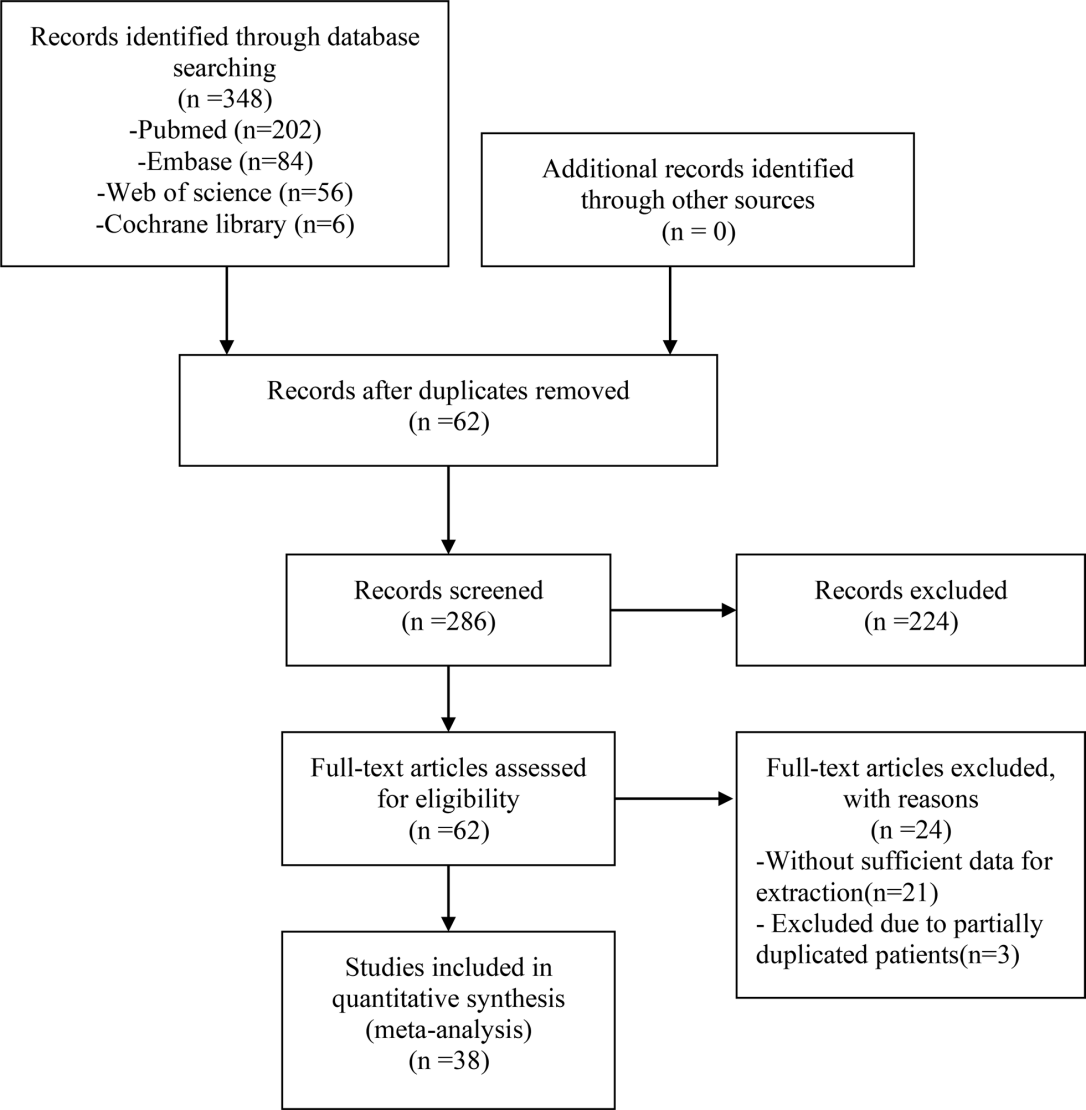
The flow chart shows the selection of studies for meta-analysis

We preliminarily screened 348 studies from the PubMed, Embase, Web of Science, and Cochrane Library databases. After reading the articles, 310 studies did not conform to the inclusion criteria. Therefore, 38 studies [1–38] were finally included in the meta-analysis. All the included studies were retrospective and had evidence of 3B or 4 according to the criteria of the Center for Evidence-Based Medicine in Oxford, UK. All observation studies had a quality score of 5 or greater on the Newcastle–Ottawa scale and were considered to have high quality (Fig. 1; Table 1).

**Table 1:** Characteristics of the included studies

Study	Year	Time frame	Level of evidence^a^	Quality score^b^	Country	Age^c^ (years)	Total pts. (n)	Male	Female	Median follow-up (months)	Fixation failure rate (%)
McLain, R. F	1991	1984–1989	4	7	USA	49.55	11	7	4	17	36.36
Dickman, C. A	1992	1987–1991	4	7	USA	47	104	55	49	20	17.31
Rompe, J. D	1993	1987–1991	4	7	Germany	61	50	23	27	≥ 12	6.45
Bilsky, M. H	2002	1985–1999	4	7	USA	54	42	28	14	35	4.76
Vrionis, F. D	2003	2000–2003	4	7	USA	53	96	56	40	NA	3.10
Mazel, C	2004	1994–2000	4	7	France	52	34	27	5	15	5.88
Villavicencio, A. T	2005	1993–1999	4	6	USA	51	58	NA	NA	NA	3.50
Bilsky, M. H	2005	1996–2003	4	7	USA	53	41	22	19	NA	7.32
Street, J	2007	NA	4	6	Canada	NA	96	NA	NA	NA	1.04
Placantonakis, D. G	2008	1996–2006	4	7	USA	52	90	58	32	21	12.00
Stevens, Q. E	2009	2003–2006	4	7	USA	56.3	34	17	17	12	5.88
Matsumoto, M	2011	1997–2009	4	7	Japan	46.5	15	12	3	41.5	40.00
Rajpal, S	2012	1995–2009	4	7	USA	56.3	37	20	17	21	2.70
Jandial, R	2013	2008–2010	4	7	USA	56.64	11	6	5	14	9.09
Matsumoto, M	2013	1997–2009	4	7	Japan	55.3	8	5	3	76.8	37.50
Yoshioka, K	2013	2006–2012	4	7	Japan	49.6	26	11	15	26.5	3.85
Bellato, R. T	2015	2009–2014	4	7	Brazil	56.71	105	54	51	7.4	8.57
Luzzati, A. D	2015	1994–2011	4	7	Italy	48	38	18	20	39	2.60
Mesfin, A	2015	2001–2013	4	7	USA	50.7	10	9	1	NA	10.00
Sellin, J. N	2015	1993–2010	4	7	USA	59	43	26	17	NA	4.65
Boriani, S	2016	1990–2015	4	7	Italy	44.1	216	113	103	45	10.19
Glorion, M	2016	1992–2004	4	7	France	45.9	88	60	28	49.4	9.09
Goodwin, C. R	2016	2004–2014	4	6	USA	NA	21	NA	NA	51	38.10
Sciubba, D. M	2016	2004–2014	4	7	USA	47	23	15	8	50	39.10
Pedreira, R	2017	2003–2013	4	7	USA	60/65	159	85	74	≥ 3	1.90
Scotto, G	2017	1992–2017	4	7	Italy	NA	518	NA	NA	NA	5.10
Shah, A. A	2017	2010–2016	4	7	USA	58	33	20	13	18	25.00
Yoshioka, K	2017	2006–2010	4	7	Japan	53.3	47	20	27	71.3	17.00
Shimizu, T	2018	1993–2015	4	7	Japan	38	30	13	17	87	20.00
Sugita, S	2018	1992–2008	4	6	Japan	63	191	NA	NA	9.9	27.00
Barzilai, O	2019	2016–2017	4	8	USA	63.5	53	30	23	4.93	6.00
Barzilai, O	2019	2010–2015	4	7	USA	61	88	44	44	44.6	12.50
Park, S. J	2019	2002–2015	4	7	Korea	49	32	18	14	49.8	37.50
Li, Z. H	2020	2009–2017	4	7	China	37.1	30	20	10	41.8	26.67
Park, S. J	2020	2010–2017	4	6	Korea	NA	136	NA	NA	16.5	6.62
Shinmura, K	2020	2010–2015	4	7	Japan	NA	61	NA	NA	> 24	42.60
Wei, H. Y	2020	2015–2018	4	7	China	45.5	15	7	8	31.1	6.67
Wong, Y. C	2020	2007–2017	4	7	China	57.3	88	45	43	NA	10.20

^a^Level of evidence: according to the criteria of the Centre for Evidence-Based Medicine

^b^Quality score: the score of the study using the Newcastle–Ottawa Scale

^c^Age is represented by the median or the average age of the study population

### Spinal fixation mechanical failure rate

**Fig. 2:**
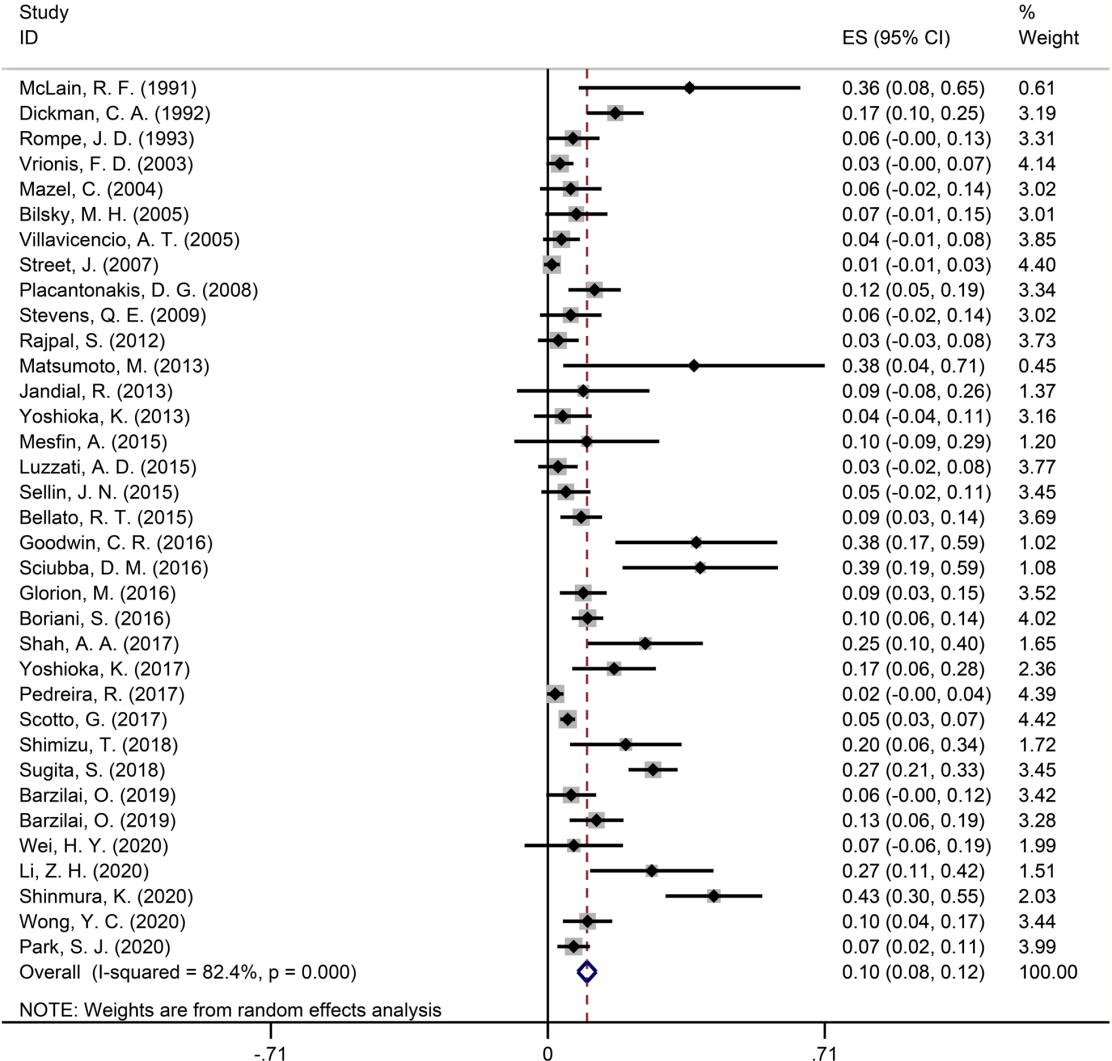
Forest plot showing the pooled spinal fixation mechanical failure rate

The pooled data on the spinal fixation mechanical failure rate consisted of 35 [1–3, 5–10, 12–37] studies with 2689 patients. The pooled failure rate was 10% (95% CI 8–12%) and is shown in Fig. 2.

The prognostic factors with similar variables were pooled in the meta-analysis. The details of the meta-analysis results are shown in Table 2.

**Table 2:** Show results of meta-analysis including pooled OR, 95% CI, sensitivity analysis, and publication bias

Prognostic factors	N	OR range	Pooled OR	Pooled 95% CI	Heterogeneity (*I*^2^) (%)	Model	*p*	Sensitivity analysis	Affected study	Publication bias (Egger’s test)
The older versus the younger [3–6, 8, 11, 18]	7	0.73–2.55	1.01	0.97–1.05	0.0	Fixed	0.634	No effect	None	0.634
The female versus the male [3–6, 8, 11, 18]	7	0.44–12.75	1.17	0.67–2.04	0.0	Fixed	0.591	No effect	None	0.455
With versus without chemotherapy [3, 6, 11, 18]	4	0.19–2.59	1.77	0.83–3.78	8.8	Fixed	0.142	Effect	Matsumoto, M	0.183
With versus without radiotherapy [3–6, 8, 11, 18]	8	0.09–10.89	2.56	0.99–6.62	56.0	Random	0.053	No effect	None	0.894
Primary versus metastatic tumor [3, 4, 11]	3	0.57–2.13	0.93	0.46–1.87	0.0	Fixed	0.834	No effect	None	0.750
Thoraciclumbar versus thoracic level [4, 5, 11]	3	1.75–4.25	2.26	1.07–4.77	0.0	Fixed	0.032	No effect	None	0.642
Lumbar versus thoracic level [3–5, 11]	4	1.40–7.29	2.49	1.37–4.53	0.0	Fixed	0.003	No effect	None	0.890
Posterior only versus combined approach [3, 11]	2	1.14–4.76	1.46	0.47–4.50	0.0	Fixed	0.514	Effect	Matsumoto, M	–
Multiple versus single vertebrae resection [3–5, 8, 11, 18]	6	0.22–2.10	0.97	0.48–1.94	0.0	Fixed	0.930	No effect	None	0.726
thin versus thick rod [4, 11]	2	1.08–1.50	1.27	0.54–2.96	0.0	Fixed	0.587	No effect	None	–
Longer versus shorter constructed length [3, 8, 11, 18]	4	0.91–3.25	1.13	0.79–1.61	47.9	Fixed	0.498	Effect	Wong, Y. C	0.365
With versus without cage subsidence [5, 11]	2	4.05–14.63	5.46	1.48–20.17	0.0	Fixed	0.011	No effect	None	–

### Age

Seven studies [3–6, 8, 11, 18] compared the spinal fixation failure rate between the older and younger subgroups. Values of *I*^2^ = 0.0% and *p* = 0.945 were obtained after the OR values of the failure rate were merged, indicating that no heterogeneity existed. A fixed-effect model was used to merge the data (OR = 1.01, 95% CI 0.97–1.05 and *p* = 0.634), showing no significant difference in the failure rate between the older and younger subgroups.

### Sex

Seven studies [3–6, 8, 11, 18] comparing the failure rate between males and females were included. Values of *I*^2^ = 0.0% and *p* = 0.694 were obtained after OR values of failure rate were merged, indicating that no heterogeneity existed. A fixed-effect model was used to merge the data (OR = 1.17, 95% CI 0.67–2.04 and *p* = 0.591), suggesting that the failure rate did not significantly differ based on sex.

### Chemotherapy

Four studies [3, 6, 11, 18] evaluated chemotherapy as a risk factor for spinal fixation failure. Values of *I*^2^ = 8.8% and *p* = 0.349 were obtained after OR values of failure rates were merged, indicating that no heterogeneity existed. A fixed-effect model was used to merge the data (OR = 1.77, 95% CI 0.83–3.78 and *p* = 0.142). The results showed no significant difference in the failure rate between patients who received chemotherapy and those who did not receive chemotherapy.

### Radiotherapy

A total of 8 studies (including subgroups) [3–6, 8, 11, 18] assessed the association between radiotherapy and failure rate. Values of *I*^2^ = 56.0% and *p* = 0.415 were obtained after the OR values of the failure rate were merged, indicating that heterogeneity existed. The pooled result via a random-effects model minimally indicated that patients with radiotherapy had a higher risk of fixation failure than patients without radiotherapy (OR = 2.56, 95% CI 0.99–6.62, *p* = 0.053).

### Tumor histology

Three studies [3, 4, 11] evaluated the relationship between tumor histology and failure rate. Values of *I*^2^ = 0.0% and *p* = 0.541 were obtained after the OR values of the failure rate were merged, indicating that heterogeneity did not exist. Thus, a fixed-effect model was applied. No significant difference in tumor histology was observed (OR = 0.93, 95% CI 0.46–1.87, *p* = 0.834).

### Tumor site

Four studies [3–5, 11] evaluated the relation between the tumor site and failure rate. Three studies [4, 5, 11] compared the failure rate between thoracic-lumbar and thoracic levels with no heterogeneity (*I*^2^ = 0.0% and *p* = 0.972). Thus, a fixed-effect model was applied. Thoraciclumar level had an increased risk of fixation failure (OR = 2.26, 95% CI 1.07–4.77, *p* = 0.032). Four studies [3–5, 11] compared the failure rate between the lumbar and thoracic levels, with heterogeneity existing (*I*^2^ = 0.0% and *p* = 0.500) and a fixed-effects model applied. Lumbar level exhibited an increased risk of fixation failure (OR 2.49, 95% CI 1.37–4.53, *p* = 0.003).

### Surgical approach

Two studies [3, 11] explored the failure rate and surgical approach included, and no heterogeneity was noted (*I*^2^ = 0.0% and *p* = 0.350). Thus, a fixed-effect model was applied. The failure rate was not significantly different based on the surgical approach (OR = 1.46, 95% CI 0.47–4.50, *p* = 0.514).

### Vertebrae resection

Six studies [3–5, 8, 11, 18] evaluated the relation between vertebrae and failure rate. Values of *I*^2^ = 0.97 and *p* = 0.671 were obtained after the OR values of the failure rate were merged, indicating that heterogeneity did not exist. Thus, a fixed-effect model was applied. A significant difference was not found in the number of vertebrae resected (OR = 0.97, 95% CI 0.48–1.94, *p* = 0.930).

### Rod diameter

Two studies [4, 11] evaluated the relation between rod diameter and failure rate. Values of *I*^2^ = 0.0% and *p* = 0.705 were obtained after the OR values of the failure rate were merged, indicating that heterogeneity did not exist. Thus, a fixed-effect model was applied. No significant difference in rod diameter was noted (OR = 1.27, 95% CI 0.54–2.96, *p* = 0.587).

### Constructed length

Four studies [3, 8, 11, 18] included the failure rate and constructed length. No heterogeneity was noted (*I*^2^ = 47.9% and *p* = 0.124), and a fixed-effect model was applied. The meta-analysis failed to find significance among different constructed lengths (OR = 1.13, 95% CI 0.79–1.61, *p* = 0.498).

### Cage subsidence

Two studies [5, 11] evaluated the relation between cage subsidence and failure rate. Values of *I*^2^ = 0.0% and *p* = 0.416 were obtained after the OR values of the failure rate were merged, indicating that heterogeneity did not exist. Thus, a fixed-effect model was applied. Collectively, cage subsidence is a significant risk factor for spinal fixation failure (OR = 5.46, 95% CI 1.48–20.17, *p* = 0.011).

### Sensitivity analysis and publication bias

Sensitivity analysis was performed in these groups. The pooled OR of chemotherapy changed significantly when excluding the study by Matsumoto [11]. The pooled OR of the surgical approach changed significantly when excluding the study by Matsumoto [11]. The pooled OR of constructed length changed significantly when excluding the study by Wong [8]. The results of the other meta-analysis did not change after removal of any one study.

Egger’s test was completed to examine the existence of publication bias. Publication bias failed to evaluate the surgical approach, rod diameter and cage subsidence because these subgroups only included two studies. Egger’s test resulted in *p* ≥ 0.05 in the other groups and indicated that the possibilities of publication bias can be excluded.

## Discussion

Durable reconstruction is required to achieve spinal stabilization after tumor resection [3, 4]. Fixation failure is a troubling complication for tumor patients who acquire long-term survival with effective therapy [10–12]. Therefore, it is important to identify risk factors affecting spinal fixation and optimize reconstruction proposals. In this study, we performed a systematic review and meta-analysis to evaluate the failure rate of spinal fixation after tumor resection and to investigate the related risk factors for spinal fixation failure.

Although complications, including fixation failure, have been reported in numerous studies, the incidence varies. Thus, the practical fixation failure rate remains unclear. Sciubba et al. [18] studied 23 patients who underwent TES of the lumbar spine and reported that 9 (39.1%) patients experienced instrumentation failure. Luzzati et al. [38] studied 38 patients with multilevel TES for tumors of the thoracic and lumbar spine and found that only one (2.6%) patient required revision of instrumentation secondary to mechanical failure. Boriani et al. [13] reviewed 220 cases treated by TES in the spine and reported that hardware failure occurred in 22 (10%) cases. Mesfin et al. [28] assessed 10 patients with TES for primary and secondary spinal tumors, and 1 (10%) patient experienced hardware failure and required revision. In this study, the incidence of spinal fixation failure was 10% (range 8–12%), which eliminated the heterogeneity caused by different sample sizes in these studies.

### Radiotherapy

The quality and strength of bone are influenced by radiation, which may affect the stabilization of spinal fixation. Matsumoto et al. [11] reported that all 3 patients with preoperative radiotherapy suffered hardware failure, whereas only 3 of the 12 patients without preoperative radiotherapy suffered instrumentation failure. Li et al. [3] found that perioperative radiotherapy was associated with instrumentation failure and reported that radiation may not only influence vertebral bone quality but also lead to muscle atrophy and weakness. However, Wong et al. reported the opposite result. Specifically, radiotherapy reached statistical significance with fixation failure being less likely to develop following radiation. They believed that vertebral recalcification occurring after radiotherapy could increase the load-sharing ability of the vertebra, which may explain the reduced implant failure rate after radiotherapy [8]. In our study, there was a trend to indicate that radiotherapy may represent a risk factor for spinal fixation failure.

### Tumor level

Regarding tumor location, Matsumoto et al. [11] failed to indicate that tumor level was significantly related to instrumentation failure. However, Yoshioka et al. reported that the resection level was a risk factor for fixation failure after multilevel TES and considered that an upper spinal level promotes better stability than a lower spinal level due to the lower exposure to mechanical stresses. In addition, there were disadvantage factors for the lower spinal level, including the long resection length and spinal instability caused by the mobility of the thoracolumbar and lumbar levels [5]. Park et al. [4] reported that TES at the lumbar level had the highest risk of instrumentation failure followed by thoracolumbar and thoracic levels, and explained that the lumbar spine has the greatest moment of flexion force and lacks adjacent stabilizing structures, such as ribs of the thoracic spine. In our study, we found that the tumor level was a risk factor for spinal fixation failure, which was consistent with most of the literature.

### Cage subsidence

Matsumoto et al. mentioned that cage subsidence resulted in the failure of loading sharing in the anterior spinal column, leading to an increased force imposed on the posterior fixation. In this study, they reported that cage subsidence was significantly related to instrumentation failure [11]. However, Yoshioka et al. [5] did not find a relationship between cage subsidence and instrumentation failure and insisted on the importance of eventual bony fusion, which prevented instrumentation failure despite cage subsidence. Our study found that cage subsidence is one of the reasons for fixation failure.

### Limitations

This meta-analysis had some limitations. First, our meta-analysis was based on retrospective studies, so selection bias was possible. Second, prognostic factor analysis included some studies with small samples, which might result in publication bias and affect sensitivity. Further studies may be needed to verify our conclusions. Furthermore, the follow-up time varied in each study. Despite these limitations, this study applied a series of measures and strict standards to evaluate the quality of these studies.

## Conclusion

In conclusion, our results indicate that the spinal fixation mechanical failure rate was 10%. Spinal fixation failure is mainly associated with tumor level, cage subsidence and radiotherapy. Durable reconstruction is needed for patients with these risk factors.

## Data Availability

Please contact the authors for data requests.
